# Deforestation effects and house invasion by chagas disease vectors in Brazil

**DOI:** 10.1038/s41598-025-22086-z

**Published:** 2025-10-31

**Authors:** Gilmar Ribeiro-Jr, Mariane Reis Vila Verde, Hernan Darío Argibay, Cristiane Wanderley Cardoso, Fabiano Simões, Eliaci Couto de Lima Costa, Cristiane Medeiros Moraes de Carvalho, Renato Barbosa Reis, Marcia C. Castro, Rodrigo Gurgel-Gonçalves, Mitermayer G. Reis

**Affiliations:** 1 Laboratory of Pathology and Molecular Biology, Gonçalo Moniz Institute - Oswaldo Cruz Foundation, Rua Waldemar Falcão, 121, Candeal - Salvador/BA, Bahia, 40296-710 Brazil; 2 Translational Program in Chagas Disease of Fiocruz (Fio-Chagas), Oswaldo Cruz Foundation – Rio de Janeiro (FIOCRUZ-RJ), Rio de Janeiro, Brazil; 3 Salvador University - Unifacs, Salvador, 41820-020 Brazil; 4 Strategic Information Center for Health Surveillance (CIEVS), Department of Salvador, Municipal Health, Salvador, Brazil; 5 Zoonosis Control Center – CCZ, Rua Mocambo, s/n, Trobogy – Salvador/BA, Salvador, 41301- 110 Brazil; 6 Epidemiological Surveillance Directorate of the State of Bahia, Bahia State Department of Health, Salvador, Bahia Brazil; 7 Department of Global Health and Population, Harvard T.H. Chan School of Public Health, Boston, MA 02446 USA; 8 Laboratory of Medical Parasitology and Vector Biology, Faculty of Medicine, University of Brasília - UNB, Darcy Ribeiro University Campus, Asa Norte – Brasília/DF, Brasília, 70910-900 Brazil; 9 Faculty of Medicine, Federal University of Bahia, Rua Reitor Miguel Calmon, s/n, Vale do Canela – Salvador/BA, Salvado, 40110-100 Brazil; 10 Yale University, 06520 New Haven, Connecticut, USA

**Keywords:** Chagas disease, Triatomine, Deforestation, Atlantic forest, One-health, Urban ecology, Computational biology and bioinformatics, Risk factors

## Abstract

We analyzed how deforestation affects triatomine presence and distribution in Salvador, Bahia, Brazil, from 2007 to 2019. Using land change data from MapBiomas (1985–2022), we assessed land cover shifts, including forest, deforestation, and urbanization, and their impact on *Trypanosoma cruzi* vectors. Urban growth has largely replaced mixed-use and forest areas, bringing some populations closer to remnants of the forest and increasing triatomine presence indoors. We documented 1,518 triatomine records, with ~ 50% testing positive for *T. cruzi*. Statistical models showed that deforestation and the remaining forest patches within neighborhoods are key factors influencing triatomine frequency. This urban expansion raises the risk of parasite transmission among vulnerable groups. To mitigate this, we recommend enhanced surveillance, control strategies, and health education in high-risk neighborhoods, aiming to reduce exposure and prevent disease transmission associated with deforestation and habitat changes.

**Supplementary Information:**

The online version contains supplementary material available at 10.1038/s41598-025-22086-z.

## Introduction

 Human Chagas disease (HCD) is a medical condition with high social and individual impact, affecting human populations across the American continent, with repercussions felt globally^1^. In HCD, as with other vector-borne tropical diseases, the socioeconomic and environmental factors influence the outcome and impact of the condition^2,3^. Historically, HCD has been broadly associated with poverty and precarious housing conditions, with *Trypanosoma cruzi* transmission to humans mainly occurring in rural areas, through the classical vectorial route, which involves competent triatomine species in colonizing inside households^4,5^. However, because of anthropogenic migratory phenomena from rural to urban areas^6^ and land occupation in these regions, the epidemiological profile of Chagas disease has been undergoing strong modifications in many Latin American areas^7^. Recent research has shown that synanthropic triatomine in the household environment poses a risk of *T. cruzi* transmission to humans and domestic animals, even in large urban centers. This transmission can occur either through the classical vectorial route or oral-vectorial transmission^8^.

Vector-borne transmission of *T. cruzi* in urban areas could be associated with flying triatomines from forest patches that often invade houses^9,10^. If infected, triatomine insects invading residences, attracted by various factors, including artificial light, can transmit *T. cruzi*^11,12^. Consequently, recurrent cases of oral-vectorial transmission have been described throughout the American continents, including Brazil and Bahia state^13,14^.

To understand how anthropogenic landscape processes influence natural ecosystems and their association with human pathogen vectors at a local scale, an alternative is the use of remote sensing images obtained through satellites^15,16^. Satellite data provides a good indicator of vegetation and environmental anthropic disturbance, which directly influences entomological fauna and diseases transmitted by insects, such as triatomines and Chagas disease^17^. Thus, the Brazilian MapBiomas project has contributed to developing a rapid and reliable method for processing large-scale and low-cost geospatial datasets and generating land coverage and transition maps. The information set used in the MapBiomas project was obtained from the Landsat Thematic Mapper™, Enhanced Thematic Mapper Plus (ETM+), and the Operational Land Imager and Thermal Infrared Sensor (OLI-TIRS) sensors on Landsat 5, Landsat 7, and Landsat 8, respectively^18^. This allows for temporal and comparative analyses of information from micro- to macro-scales.

In this study, we describe the effect of human occupation of natural landscapes on the frequency and spatial distribution of *T. cruzi* vectors and identify the vulnerable areas to *T. cruzi* vectorial transmission in a Brazilian urban Atlantic Forest area.

## Results

### Land occupation and use in the City of Salvador

**Fig. 1:**
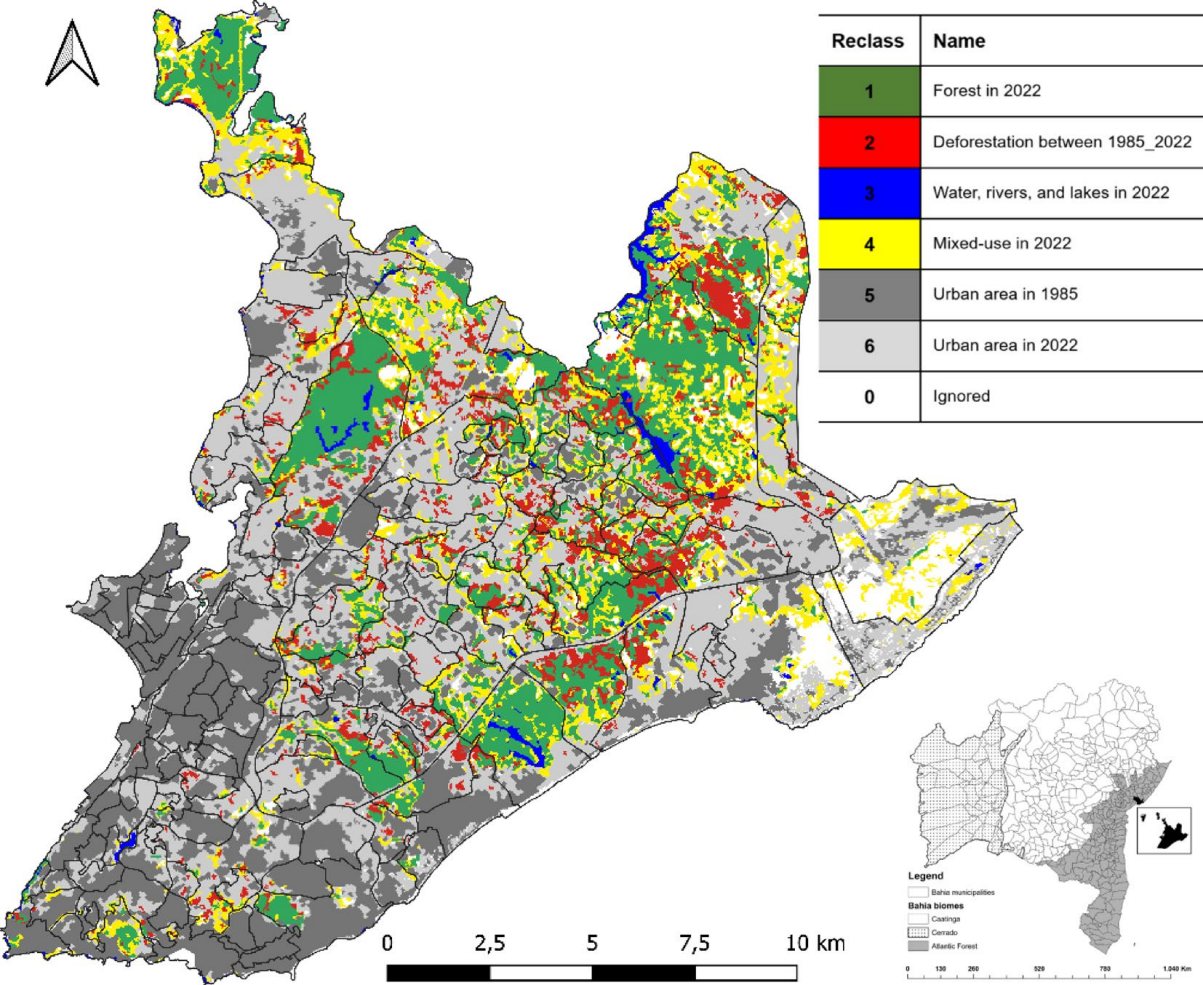
Study area. Biomes of Brazil and geolocation of the state of Bahia (left). Municipalities of Bahia (right); the detail shows the capital, the city of Salvador. Legend: Land cover transition graph of Salvador between 1985 and 2022. Source: MapBiomas, collection 8.0.

**Fig. 2:**
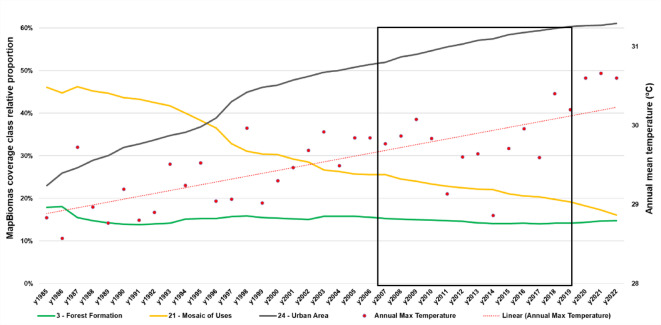
Land cover transition of the main variables analyzed and annual mean temperature for Salvador between 1985 and 2022. Data: MapBiomas, collection 8.0. The box in detail shows the study period, and the red line means a linear tendency model of this data.

The patterns of land occupation and land use in the City of Salvador were mainly described by the transition between the Urban Area and the Mosaic of Mixed Use (class 21), as shown in Fig. 1. In the MapBiomas^®^ project, the class “Mosaic of Uses” is defined as “agricultural areas where it was not possible to distinguish between pasture and agriculture”^19^. From 1985 to 2022, the proportion of Urban Areas (class 24) in the City of Salvador nearly tripled, increasing from 23% to 61%. Over the same period, Forest formations (class 3) decreased from 17.9% to 14.7%, while Rivers and Lakes (class 33) decreased from 2.89% to 1% in their proportion (Fig. 2). The transition for all MapBiomas^®^ classes can be seen in Supplementary Material 1.

Regarding the location of the main remnants of the Forest Formation class, the major fragments are isolated from each other and distributed mostly in the northern and eastern regions of the city of Salvador. In this regard, it is worth highlighting the importance of public parks, the Brazilian Armed Forces, and municipal protected areas for historically maintaining these fragments, emphasizing the necessity for a consolidated set of laws to protect this heritage.

**Fig. 3:**
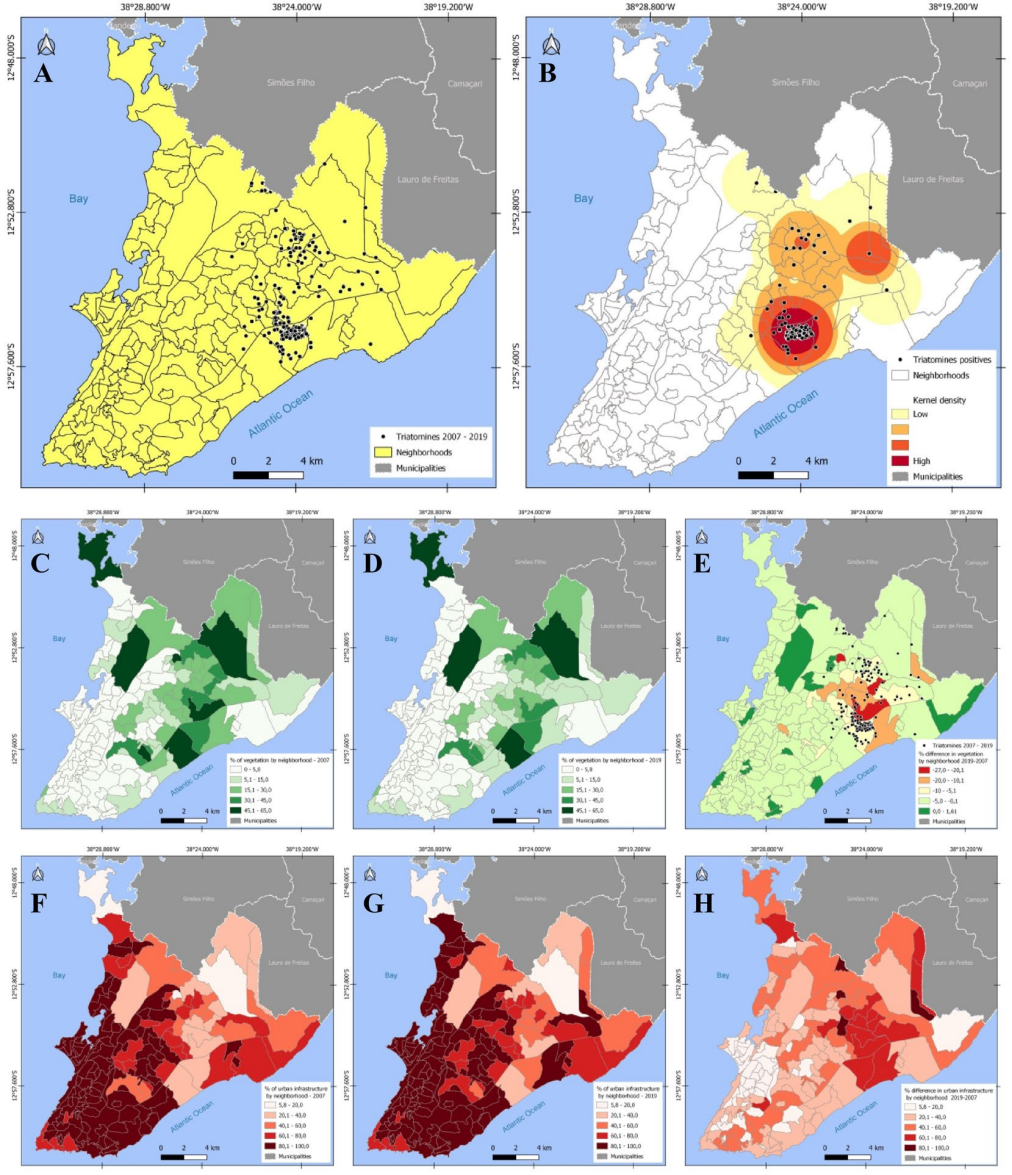
(**A**) Triatomines 2007–2019, (**B**) *T. cruzi*-infected triatomines, and geolocation stratified by neighborhood of the (**C**) Forest in 2007, (**D**) 2019 and (**E**) Deforestation between 2019 − 2007, (**F**) Urban Area 2007, (**G**) 2019, and (**H**) Urban Area between 2019 − 2007.

Figure 3 displays the geolocation of the descriptive variables analyzed: Triatomine data from 2007 to 2019, Forest Formation in 2019, Deforestation between 2007 and 2019, Urban Area in 2019, and Population in 2019, stratified by neighborhood.

## Description of environmental data and population indicators

The city of Salvador has an area of 692.818 square kilometers and is administratively divided into 160 neighborhoods. In 2007, the city displayed 18.45% (51,473,447.77 square meters) of its area classified as Forest Formation, which was reduced by approximately 3% (8,084,436.09 square meters) in 2019. Conversely, the proportion of Urban Area, which was 62.49% (174,303,097.49 square meters) in 2007, increased by 6.24% (17,417,904.61 square meters) by 2019. The stratification of descriptive variables by neighborhood can be seen in Fig. 3, with detailed data and code descriptions available in Supplementary Material 3.

## Entomological evaluation

A total of 1,518 triatomines were reported during the period from 2007 to 2019, and geographic coordinates were obtained for 1,511 (99.53%) of these records Fig. 3. Among the reported triatomines, the majority (99.53%, *n* = 1511) were *Panstrongylus tibiamaculatus* (Bittinelli, 2022)^20^, while the remaining 0.47% (*n* = 7) were identified as *Panstrongylus geniculatus* (Latreille, 1811). Because of the significant representation of *P. tibiamaculatus*, subsequent analyses were conducted only with data from this species.

Of the 1,511 specimens of *P. tibiamaculatus* collected, 517, 698, and 296 specimens were collected in the intra-, peri-, and extra-domicile environments, respectively. Almost half of the adult triatomines were females, and ~ 50% of them were infected by *T. cruzi* through the parasitological examination. The triatomines recorded in the intra-domicile were all adult specimens, and there was no record of colonization of the intra- or peri-domicile. However, it is worth noting that in many cases, the peri-domicile of residences is the continuation of the natural vegetation of the wild environment.

The analysis of triatomine notifications revealed a highly clustered and annual recurrent spatial distribution throughout the study period, particularly in neighborhoods characterized by significant forest areas and deforestation. Specifically, 78.97% of the triatomine (*n* = 1,199) were reported from the neighborhood with the largest Forest formation areas.

## Determinants of triatomine frequency and Spatial distribution

The implemented models indicated that deforested areas was the most influential factor explaining neighborhood-level variation in reported triatomine in the city of Salvador (Table 1). The INLA-BYM model with all covariates showed a positive and statistically significant association between Deforestation and the reported triatomine counts (Incidence Rate Ratio, IRR: 3.29; 95% CI: 1.43–7.64) (Table 2). The ridge regression approach, treating both landscape predictors as IID random effects with PC priors (pc.prec(1,0.01)), improved fit substantially (DIC = 250.66, WAIC = 253.83). In that specification, the posterior precision for Forest area was relatively low (precision ≈ 0.45,), indicating substantial between-area variability associated with forest, whereas the posterior precision for Deforested area was higher (precision ≈ 8.39), meaning the deforestation term was largely shrunk toward zero when modelled as an iid effect (Table 2 and Sup. Material table S1).

**Table 1:** Multivariable zero-inflated negative binomial INLA-BYM model of triatomine counts per neighborhood according to forest, deforested area cover, and human inhabitants.

	IRR (95% CI)
(Intercept)	3.18 (0.89–11.48)
Population (1000 hab)	0.94 (0.90–0.99)
Forest area (Km2)	2.19 (0.49–12.66)
Deforested area (Km2)	3.29 (1.43–7.64)

**Table 2:** Results of the INLA-BYM zero-inflated negative binomial model with ridge regression for triatomine abundance. Estimates of fixed effects (Population and deforested area) are presented as posterior mean and 95% credible interval (0.025quant – 0.975quant). The random effects include independent and identically distributed (IID) priors for forest area to account for collinearity through ridge regression, as well as a Besag-York-Mollié (BYM) model for Spatial effects. The model hyperparameters include precision estimates for random effects and the zero-inflation component of the negative binomial distribution.

Fixed effects	mean	0.025quant	0.975quant
(Intercept)	-1.50	-2.91	-0.22
Population (1000 hab)	-0.06	-0.11	-0.01
Deforested area (Km2)			
**Model hyperparameters**			
size for nbinomial_1 zero-inflated observations	1265.09	2.87	7944.53
zero-probability parameter for zero-inflated nbinomial_1	0.173	0.038	0.421
Precision for the Forest area	0.219	0.122	0.359
Precision of the IID component	2219.266	158.71	8541.932
Precision of spatial component	2246.76	153.499	8825.007

To explore alternative allocations of variation, we also fitted models with one predictor fixed and the other random (Sup. Material table S1). When Forest area was fixed and Deforested area specified as an IID effect, the posterior precision for Deforested area was low (precision ≈ 0.44), indicating substantial residual heterogeneity linked to deforestation. Conversely, when Deforested area was fixed and Forest area modelled as IID with PC prior (pc.prec(1,0.01)), the best-fitting specification in our comparison, model fit improved further (DIC = 228.38, WAIC = 219.64). In this preferred model, Deforested area was strongly and positively associated with triatomine counts (fixed effect mean = 5.44, 95% CI: 2.91–10.79), while Forest area entered as a random effect with posterior precision ≈ 0.219 (posterior 95%CI for precision: 0.122–0.360), i.e. a relatively low precision corresponding to notable between-area variability attributable to forest-related heterogeneity. Together, these results indicated that: (1) deforestation acted as a consistent, direct predictor of triatomine counts and was best represented as a fixed effect, while (2) forest area captured heterogeneous between-neighborhood variation better modeled as a random effect, and (3) ridge-type regularization with PC priors effectively distinguished predictors contributing systematic effects from those reflecting spatially structured heterogeneity.

## Discussion

This study describes the transitions in land cover and uses that occurred in Salvador between 1985 and 2022 and investigates how the Atlantic Forest landscape influences the frequency and spatial distribution of *T. cruzi* vectors between 2007 and 2019. We apply a mixed-approach method to evaluate entomological and environmental information, identifying vulnerable areas and exposed populations to vectorial and oral *T. cruzi* transmission within the study area. We also describe statistically significant associations between the frequency and spatial distribution of triatomine and landscape metrics of an Atlantic Forest area in Brazil.

Our findings indicate that deforestation is a consistent and robust predictor of reported triatomine counts across neighborhoods, whereas forest cover reflects more heterogeneous, area-specific variation. The ridge-type regularization further helped to disentangle collinear predictors, demonstrating that the deforested area is best treated as a fixed effect with a strong positive association with triatomine counts, while forest area is more appropriately modeled as a random effect capturing unstructured heterogeneity. Together, these results highlight that deforestation processes represent a direct ecological driver of triatomine presence, whereas remaining forest may reflect local habitat-specific contexts not fully captured by global fixed effects.

Chagas disease has historically been associated with poverty, adobe houses, rural and isolated areas, and a low human development index^21^. However, the rapid urbanization process in many Latin American cities has led to extensive urban territorial expansion for human habitation. This expansion has resulted in the deforestation and fragmentation of different biomes, including the Atlantic Rainforest^22^, increasing the exposure of these populations to different zoonotic diseases, including Chagas disease.

The Brazilian Atlantic Rainforest biome originally occupied an extensive area along the east coast, spanning 17 states from Rio Grande do Norte to Rio Grande do Sul. However, due to logging, agricultural expansion, urbanization, and other anthropogenic activities, over 90% of this original Atlantic Forest coverage has been lost over the last five centuries^23^. Deforestation and fragmentation of natural biomes cause habitat loss for many animal and plant species^24^. Among the affected biological entities are some native triatomines, which have become increasingly present by colonizing human households in both urban and rural areas of Bahia^25,26^.

Triatomines are naturally wild insects that occur in diverse ecological niches and play a well-defined role in the micro-predation of various vertebrate species, including, occasionally, humans and their domestic animals^27^. Some species of triatomine have populations capable of adapting to anthropic environments and approaching human populations through a process known as colonization. On the other hand, some triatomine species are typically wild and rarely colonize domestic environments, being found intrusively inside residences as winged adults^28,29^. Leite et al. (2011)^30^ tested the hypothesis that house invasions by sylvatic triatomine in the Atlantic Forest are stimulated by environmental degradation. They suggest that less degraded areas maintain larger populations of triatomines, consequently presenting higher indices of dispersal and domiciliary invasion. Our results support this hypothesis since the multivariable models we implemented fit well with the neighborhoods of Salvador that have a high proportion of Atlantic Forest remnants. By applying ridge regression, we demonstrated that deforestation is the dominant environmental driver of triatomine presence in the households, whereas vegetation coverage alone does not have an independent effect when both factors are modeled together.

Other factors can contribute to sylvatic vectors invading houses, such as light incidence, once triatomine has a positive phototropism behavior^11,12^. Artificial light in houses near forest remnants could attract triatomines, allowing house invasion. Lastly, global warming can significantly increase *T. cruzi* exposure once warmer temperatures enhance triatomine offspring and reduce the lifecycle^31^. Due to this, there is a need for modeling data and developing predictive models for defining *T. cruzi* vulnerable areas in space and time^31^.

Both colonizing and invading triatomine species play an important role in *T. cruzi* transmission in domestic environments, with an increased risk associated with colonizing species. In this study, the natural *T. cruzi* infection rate of *P. tibiamaculatus* was ~ 50%, determined through parasitological methods. This relatively high natural infection rate is likely common for wild species of triatomine, especially those with intrusive behavior in the domestic environment^32^. Winged adults must go through five nymph stages, requiring several blood meals from sylvatic animals (*T. cruzi* reservoirs) in their natural habitat before becoming adults and developing wings. This increases the likelihood of the triatomine being infected when they invade homes. Consequently, the presence of *T. cruzi*-infected triatomines inside houses indicates an increased risk of parasite exposure in urban and rural environments, highlighting the need for comprehensive health education efforts to reduce *T. cruzi* exposure in vulnerable populations.

In our case, the encounter of *P. tibiamaculatus* and *P. geniculatus* is increased in communities near the Forest formations of the Atlantic Forest biome or areas resulting from the deforestation of these forest fragments. These communities represent the vulnerable populations and areas at risk for *T. cruzi* transmission in Salvador city. Additionally, despite no records of human blood meals and only two records of nymphs inside houses by *P. tibiamaculatus* in Salvador^25^, risk of *T. cruzi* transmission through the vectorial-oral route exists due to the presence of infected triatomines inside houses.

The persistent invasion of houses by *P. tibiamaculatus* in urban settings increases the risk of vector/oral transmission of *T. cruzi*. The presence of urban vectors of Chagas disease in the American continent was reviewed^33^ showing that 18 species have been found in urban settings mainly *Triatoma* and *Panstrongylus* in all types of cities from Argentina to the USA. Urban vectors of *T. cruzi* are challenging, and their surveillance and control will require new strategies to be developed by health systems. Innovations have been suggested to strengthen surveillance with community participation such as online training for health agents, development of apps to identify vectors and improve surveillance with community, participation and citizen science, vector-borne transmission-risk mapping^34–36^. Moreover, strengthening triatomine information post ^37^could help surveillance in urban settings.

The triatomine information post (TIP), a passive strategy used to monitor *T. cruzi* vectors in Brazilian municipalities, needs special attention from the State and National levels, once it represents the start to other entomological and surveillance systems of the municipal health system, promoting evidence-based decision making at further levels. The TIP data used in this investigation can be used to monitor and predict *T. cruzi* transmission risk areas in other Atlantic Forest areas involving *P. tibiamaculatus* species, including the effects of climate change predictions among these areas. Moreover, the surveillance of vector-borne transmission of Chagas disease could be strengthened by innovative strategies with community participation ^33^.

Santos *et.* al^7^ showed that deforestation in eastern Amazonia is associated with higher odds of palm-crown infestation by *Rhodnius* vectors indicating that these bugs widely coexist with humans across disturbed landscapes. Unfortunately, these authors did not estimate the effects of deforestation on the frequency of infected vectors. In preserved forests with diverse vertebrate community, bugs might feed more frequently on poorly competent *T. cruzi* reservoirs, leading to lower infection frequency, as expected by a “dilution-effect hypothesis”^38–40^. This hypothesis needs to be tested for bugs in Amazonia and Atlantic Forest.

In conclusion, the American trypanosomiasis is naturally a zoonosis and suits a zooanthroponosis due to the proximity of human populations to the forest formations of local biomes and their wildlife. The rapid urbanization process and land use in Salvador from 1985 to 2022 occurred mainly through the replacement of the Mixed-Use and Forest formation areas, as defined by MapBiomas classes, bringing some human populations closer to Forest areas. This led to an increase in the frequency of triatomine inside household units and, consequently, an increase in the risk of *T. cruzi* transmission to these vulnerable populations. Between 2007 and 2019, 1,518 triatomine records were evaluated, with approximately 50% of them infected with *T. cruzi*. Univariate and multivariable models demonstrate that deforestation is the most important variable in explaining the count of reported triatomines per neighborhood in the city of Salvador. Although no cases of acute CD were recorded in the city of Salvador during the study period, it is recommended to strengthen the surveillance of triatomines and health education campaigns, especially in the most affected neighborhoods.

### Methods

#### Study area and design

A descriptive, retrospective, cross-sectional study, at neighborhood stratification, was conducted between 1985 and 2022 in Salvador, Bahia, Brazil, excluding the islands part of its political-geographic territory.

Salvador is the capital of Bahia state. It is located at 13ºS 38°W, with an estimated population of 2,857,329 people (the 5th largest in Brazil), and a population density of 3,859.44 inhabitants per square kilometer. Salvador covers an area of 692,818 square kilometers on the coast of the Atlantic Ocean, and its main biome is the Atlantic Rain Forest.

## MapBiomas coverage and transition data

To understand the land cover and use of the city of Salvador between the study periods, we evaluated MapBiomas land transition data from 1985 to 2022.

We evaluated each annual land cover raster from this period, and we compared the frequency of raster classes according to the MapBiomas transition classes, codes, and colors (Supplementary Material 1), respectively, using RStudio 2025.05.0 + 496 “Mariposa Orchid” (https://posit.co/download/rstudio-desktop/) for windows and QGIS version 3.42.2 (https://qgis.org/download/) software.

Then, we evaluate the land cover transitions using the available raster from MapBiomas transition, between the years 1985 and 2022. The land transition raster has codes calculated by combining the land cover codes from the first and second years, according to the formula (year1 × 100) + year2.

To achieve these classifications, Landsat satellite image files, with a resolution of 30 × 30 m, were classified using NDVI. This simple graphical indicator can analyze remote sensing measurements. The MapBiomas platform generated NDVI classification algorithms (http://mapbiomas.org/) following the method previously described by the MapBiomas Project (2024)^41^. Data generated by the MapBiomas project are available at https://brasil.mapbiomas.org/colecoes-mapbiomas, providing annual historical time series of land cover and land use transitions, as well as models of vegetation cover transition between different periods for the entire territory of Brazil and others South American countries, with data preprocessed to remove or minimize artifacts such as clouds and smoke^41^.

The Salvador vector layer (.shp) was obtained from the Brazilian Institute of Geography and Statistics (IBGE). The stratification of neighborhoods followed the information provided by the Urban Development Company of the State of Bahia (CONDER).

### Determinants of triatomine spatial distribution

We evaluated the influence of environmental and demographic indicators (descriptive variables) on Triatomine data from 2007 to 2019: Forest Formation in 2019, Deforestation between 2007 and 2019, Urban Area in 2019, and Population in 2019.

We determined the descriptive variables: Forest Formation, Deforestation, and Urban Area for the municipality of Salvador, for the period from 2007 to 2019. Estimates were made at the neighborhood level in Salvador and are described in the variable and codes dictionary (Supplementary Material 2).

- Forest: calculated as the area, in square meters, of class 03 (Forest Formation) for 2007 and 2019. Through this, we obtained the proportion of forest formation area (F) from each neighborhood: F_cl03_07/Area × 100.

- Deforestation: calculated as the difference in the proportion of forest formation per neighborhood: propF19 - propF07.

- Urban Area: calculated as the area in square meters of class 24 (Urban Infrastructure) in the years 2007 and 2019. We calculate the proportion of urban infrastructure area (IU) for each neighborhood: IU_cl24_07/Area × 100. We calculate the difference in the proportion of urban infrastructure (IU) between the years per neighborhood as propIU19 - propIU07.

- Population: The data were obtained directly from IBGE 2019 and stratified by neighborhood.

### Triatomine data

*T. cruzi* vectors were obtained by the Municipal Health Department of Salvador through the Center for Zoonosis Control (CZC-SSA). Triatomine found in household units by citizens was reported to CZC-SSA or directly to Fiocruz-BA. The triatomine was then evaluated at Insectary 1 of the Laboratory of Pathology and Molecular Biology (LPMB) - Gonçalo Moniz Institute (IGM-Fiocruz/BA), where we conducted taxonomical identification, abdominal compression examination, and fresh parasitological examination of triatomine feces between slide and coverslip to search for flagellated protozoa, *T. cruzi*-like Trypanosomatidae.

We collected the Information about each triatomine on a standardized data collection instrument, where we classified the vectors regarding, (1) address and information about where the triatomine was found at the household unit (HU): intradomicile, peridomicile, or sylvatic; (2) sex: male, female, or not suitable for analysis; (3) developmental stage: nymph or adult; (4) taxonomical identification^42^; (5) *T. cruzi* infection through parasitological evaluation^25^.

### Statistics and geoprocessing methods

After checking and reviewing the information, we compiled the dataset and raster files and descriptively analyzed them using RStudio^®^ software (version: RStudio 2024.04.0 + 735 “Chocolate Cosmos”) with Rtools44 for Windows.

For the visualization of spatial data, we used QGIS^®^ 3.26 with GRASS. The spatial unit used for mapping was the geographic coordinate point obtained through GPS (GIS) or the coordinate point obtained recursively from the address in the triatomine data form if no specific geographical coordinate information was available.

### Association between variables

We considered that the frequency of triatomine collected in residences in Salvador is influenced by the variables examined in this study, establishing a causal relationship, which is a simplification necessary for the developed research described here. The variables Forest Formation, Deforestation and Population were used to characterize and classify the neighborhoods of Salvador City.

To analyze the association between neighborhood-level descriptive variables and reported triatomine counts, we first fitted a zero-inflated generalized linear mixed model with a negative binomial distribution using the glmmTMB package^42^ in R^43^. This modeling approach is appropriate for counting data characterized by excess zeros and overdispersion. All models included population size as a covariate given its potential influence on Triatomine notifications. Model overdispersion was assessed with the DHARMa package^44^.

We then evaluated spatial autocorrelation in the residuals of the initial model to determine the need for a spatial extension. We computed local Moran’s Index and conducted a permutation test on model residuals, with the null hypothesis assuming no spatial autocorrelation and the alternative indicating positive spatial autocorrelation. We performed the spatial analyses using the Moran.mc function from the spdep package^46^.

Upon detecting significant results from the permutation test, we proceeded with a spatial model that addressed and weighed spatial autocorrelation. This was achieved using a Bayesian zero-inflated negative model with Integrated Nested Laplace Approximation (INLA)^46^ and the Besag-York-Mollié (BYM) prior to the neighborhood-level random effect.

To address multicollinearity among variables, we calculated the Variance Inflation Factor (VIF) and considered collinearity when VIF > 2. Vegetation and deforested area were initially included as fixed effects; however, VIF analysis and pairwise correlations revealed a high degree of collinearity between these two predictors, which may result in unstable coefficient estimates. To assess the robustness of covariate effects under regularization, we performed a ridge-type sensitivity analysis in which these predictors were alternatively specified as fixed effects or as iid random effects with weakly informative penalized-complexity (PC) priors on their precisions. For the IID random effects, we used PC priors of the form pc.prec(u, α); in the main analysis we set u = 1 and α = 0.01 (i.e., P(SD > 1) = 0.01), which provides moderate shrinkage toward zero while still allowing the data to dominate when informative.

 The BYM spatial component was fitted with the standard parametrization using consistent priors as implemented in INLA. Competing models were compared using Deviance Information Criterion (DIC). We report posterior means and 95% credible intervals (CI) for fixed effects, as well as posterior summaries of hyperparameters (precisions and spatial variance components). We present models including all candidate variables to provide a comprehensive assessment of predictor effects, allow robust comparison across model specifications, and retain variables with theoretical relevance, even when they do not yield the optimal fit according to performance metrics. Full model results are provided in Supplementary Material (Table S1, Figure S1).

## Supplementary Information

Below is the link to the electronic supplementary material.

Supplementary Material 1

## Data Availability

The datasets used and/or analyzed during the current study are available from the corresponding author on reasonable request.
