# Supplementary material for: Deforestation effects and house invasion by chagas disease vectors in Brazil
Source: Sci Rep. 2025 Oct 31;15:38218. doi: 10.1038/s41598-025-22086-z (PMC12579252; doi:10.1038/s41598-025-22086-z)
Supplement: Supplementary file 1 — Supplementary Material 1 [file 41598_2025_22086_MOESM1_ESM.zip › Data dictionary.docx]

**Data dictionary:**

| **Campo** | **Tipo** | **Codificação** | **Conteúdo** |
| --- | --- | --- | --- |
| Nome | Texto | - | Nome do bairro |
| Área | Numérico | #######.## | Área do bairro em metros quadrados |
| Cod_bairro | Numérico | ## | Código do bairro |
| F_cl03_07 | Numérico | #######.## | Área em metro quadrado da classe 03 (Formação Florestal) no ano de 2007 |
| propF07 | Numérico | ##.## | Proporção da área de formação florestal (F) em cada bairro: F_cl03_07/Áreax100 |
| F_cl03_19 | Numérico | #######.## | Área em metro quadrado da classe 03 (Formação Florestal) no ano de 2019 |
| propF19 | Numérico | ##.## | Proporção da área de formação florestal (F) em cada bairro: F_cl03_19/Área x100 |
| Fdifer | Numérico | ##.## | Diferença na proporção de formação florestal, por bairro: propF19 - propF07 |
| IU_cl24_07 | Numérico | #######.## | Área em metro quadrado da classe 24 (Infraestrutura Urbana) no ano de 2007 |
| propIU07 | Numérico | ##.## | Proporção da área de Infraestrutura Urbana (IU) em cada bairro: IU_cl24_07/Área x100 |
| IU_cl24_19 | Numérico | #######.## | Área em metro quadrado da classe 24 (Infraestrutura Urbana) no ano de 2019 |
| propIU19 | Numérico | ##.## | Proporção da área de Infraestrutura Urbana (IU) em cada bairro: IU_cl24_19/Área x100 |
| IUdifer | Numérico | ##.## | Diferença na proporção de Infraestrutura Urbana (IU), por bairro:  propIU19 - propIU07 |
