# Supplementary material for: Deforestation effects and house invasion by chagas disease vectors in Brazil
Source: Sci Rep. 2025 Oct 31;15:38218. doi: 10.1038/s41598-025-22086-z (PMC12579252; doi:10.1038/s41598-025-22086-z)
Supplement: Supplementary file 1 — Supplementary Material 1 [file 41598_2025_22086_MOESM1_ESM.zip › Manuscrip Supplemantary materials.docx]

**Supplementary material 1. Table S1:** MapBiomas transition codes, reclassification codes, and colors of Salvador between 1985 and 2022.

| **MapBiomas Transition class of SSA and reclass system** | | | | |
| --- | --- | --- | --- | --- |
| 303 **(1)** | 1133 **(3)** | 2124 **(6)** | 2503 **(1)** | 3321 **(4)** |
| 315 **(2)** | 1313 **(0)** | 2125 **(0)** | 2511 **(0)** | 3323 **(0)** |
| 321 **(2)** | 1321 **(4)** | 2130 **(0)** | 2515 **(0)** | 3324 **(6)** |
| 324 **(2)** | 1324 **(6)** | 2133 **(3)** | 2521 **(4)** | 3325 **(0)** |
| 325 **(2)** | 1503 **(1)** | 2149 **(0)** | 2524 **(6)** | 3330 **(0)** |
| 330 **(2)** | 1515 **(0)** | 2311 **(0)** | 2525 **(0)** | 3333 **(3)** |
| 333 **(2)** | 1521 **(4)** | 2315 **(0)** | 2530 **(0)** | 4921 **(4)** |
| 503 **(1)** | 1524 **(6)** | 2321 **(4)** | 2533 **(3)** | 4924 **(6)** |
| 505 **(0)** | 1525 **(0)** | 2323 **(0)** | 3030 **(0)** | 4949 **(0)** |
| 1111 **(0)** | 2103 **(1)** | 2324 **(6)** | 3224 **(6)** | 5023 **(0)** |
| 1121 **(4)** | 2111 **(0)** | 2325 **(0)** | 3303 **(1)** | 5024 **(6)** |
| 1124 **(6)** | 2115 **(0)** | 2423 **(0)** | 3305 **(0)** | 5050 **(0)** |
| 1125 **(0)** | 2121 **(4)** | 2424 **(5)** | 3311 **(0)** |  |
| 1130 **(0)** | 2123 **(0)** | 2430 **(0)** | 3315 **(0)** |  |

| **Reclass** | **Name** | **COD** |
| --- | --- | --- |
| **1** | Forest in 2022 | #32a65e |
| **2** | Deforestation between 1985_2022 | #d4271e |
| **3** | Water, rivers, and lakes in 2022 | #0000FF |
| **4** | Mixed-use in 2022 | #ffef08 |
| **5** | Urban area in 1985 | #757575 |
| **6** | Urban area in 2022 | #cdcdcd |
| **0** | Ignored | #000000 |

**Supplementary Material 2. Table S2: Comparison of Models for Predicting Triatomine Abundance**
The table presents results from different model alternatives for performance comparison, including models with deforested and forested areas treated as fixed, as well as models incorporating ridge-type regularization. For each model, estimated parameters, 95% credible intervals, and model fit/performance metrics (e.g., DIC, WAIC, and log-likelihood) are reported. Ridge regularization with PC priors was applied to distinguish predictors contributing systematic effects from those reflecting spatial heterogeneity.

| **Fixed effects:** | **mean** | **sd** | **0.025quant** | **0.975quant** | **Model hyperparameters:** | **8899** | **sd** | **0.025quant** | **0.975quant** | **DIC** |
| --- | --- | --- | --- | --- | --- | --- | --- | --- | --- | --- |
| (Intercept) | 1.061 | 0.565 | -0.022 | 2.194 | size for nbinomial_1 zero-inflated observations | 0.307 | 0.058 | 0.209 | 0.435 | 316.43 |
| Population  (1000 hab) | -0.051 | 0.023 | -0.097 | -0.005 | zero-probability parameter for zero-inflated nbinomial_1 | 0.627 | 0.055 | 0.512 | 0.729 |  |
| Forest area  (km2) | 3.075 | 0.565 | 1.968 | 4.184 | Precision for OBJECTID (iid component) | 3040.221 | 571.608 | 2115.589 | 4356.785 |  |
|  |  |  |  |  | Precision for OBJECTID (spatial component) | 2665.805 | 992.376 | 1297.795 | 5139.718 |  |
| (Intercept) | 1.411 | 0.624 | 0.188 | 2.618 | size for nbinomial_1 zero-inflated observations | 0.402 | 0.13 | 0.199 | 0.704 | 316.28 |
| Population  (1000 hab) | -0.062 | 0.026 | -0.112 | -0.012 | zero-probability parameter for zero-inflated nbinomial_1 | 0.656 | 0.088 | 0.463 | 0.806 |  |
| Deforested area (km2) | 1.468 | 0.289 | 0.939 | 2.072 | Precision for OBJECTID (iid component) | 2209.743 | 2419.82 | 148.032 | 8634.777 |  |
|  |  |  |  |  | Precision for OBJECTID (spatial component) | 2202.268 | 2421.381 | 145.273 | 8630.902 |  |
| (Intercept) | 1.147 | 0.663 | -0.133 | 2.436 | size for nbinomial_1 zero-inflated observations | 0.361 | 0.121 | 0.174 | 0.643 | 316.48 |
| Population  (1000 hab) | -0.058 | 0.025 | -0.108 | -0.009 | zero-probability parameter for zero-inflated nbinomial_1 | 0.618 | 0.106 | 0.389 | 0.8 |  |
| Forest area  (km2) | 0.793 | 0.826 | -0.706 | 2.552 | Precision for OBJECTID (iid component) | 2227.122 | 2543.095 | 135.46 | 8969.449 |  |
| Deforested area (km2) | 1.192 | 0.423 | 0.356 | 2.033 | Precision for OBJECTID (spatial component) | 2235.776 | 2480.907 | 148.81 | 8819.852 |  |
|  |  |  |  |  |  |  |  |  |  |  |
| Results of fitted models for Ridge test: | | |  |  |  |  |  |  |  |  |
| **Fixed effects:** | **mean** | **sd** | **0.025quant** | **0.975quant** | **Model hyperparameters:** | **mean** | **sd** | **0.025quant** | **0.975quant** | **DIC** |
| (Intercept) | 1.924 | 0.547 | 0.826 | 2.945 | size for nbinomial_1 zero-inflated observations | 38.855 | 15.451 | 17.983 | 77.723 | 250.66 |
| Population  (1000 hab) | -0.061 | 0.023 | -0.106 | -0.014 | zero-probability parameter for zero-inflated nbinomial_1 | 0.665 | 0.131 | 0.372 | 0.875 |  |
|  |  |  |  |  | Precision for Forest area (km2) | 0.447 | 0.416 | 0.062 | 1.550 |  |
|  |  |  |  |  | Precision for Deforested area (km2) | 8.387 | 0.669 | 7.018 | 9.637 |  |
|  |  |  |  |  | Precision for OBJECTID (iid component) | 2159.881 | 2065.277 | 196.757 | 7631.306 |  |
|  |  |  |  |  | Precision for OBJECTID (spatial component) | 2867.288 | 1602.409 | 1071.030 | 7099.188 |  |
| (Intercept) | 1.314 | 0.524 | 0.275 | 2.329 | size for nbinomial_1 zero-inflated observations | 67.131 | 52.346 | 10.565 | 204.173 | 253.17 |
| Population  (1000 hab) | -0.061 | 0.022 | -0.105 | -0.017 | zero-probability parameter for zero-inflated nbinomial_1 | 0.706 | 0.044 | 0.612 | 0.785 |  |
| Forest area  (km2) | 0.835 | 0.245 | 0.358 | 1.321 | Precision for Deforested area (km2) | 0.443 | 0.083 | 0.297 | 0.621 |  |
|  |  |  |  |  | Precision for OBJECTID (iid component) | 2082.793 | 1122.145 | 705.126 | 4977.668 |  |
|  |  |  |  |  | Precision for OBJECTID (spatial component) | 2432.154 | 1856.903 | 490.156 | 7331.034 |  |
| (Intercept) | -1.5 | 0.688 | -2.922 | -0.205 | size for nbinomial_1 zero-inflated observations | 800.324 | 6892.375 | 3.197 | 5310.000 | 228.96 |
| Population  (1000 hab) | -0.058 | 0.024 | -0.105 | -0.01 | zero-probability parameter for zero-inflated nbinomial_1 | 0.173 | 0.100 | 0.039 | 0.421 |  |
| Deforested area (km2) | 1.693 | 0.333 | 1.067 | 2.38 | Precision for Forest area (km2) | 0.219 | 0.061 | 0.122 | 0.360 |  |
|  |  |  |  |  | Precision for OBJECTID (iid component) | 2879.089 | 3547.500 | 228.043 | 12100.000 |  |
|  |  |  |  |  | Precision for OBJECTID (spatial component) | 2836.66 | 3502.147 | 217.566 | 11900.000 |  |

**Supplementary Material 3. Figure S1:** MapBiomas coverage classes of Salvador in 2007 and 2019.


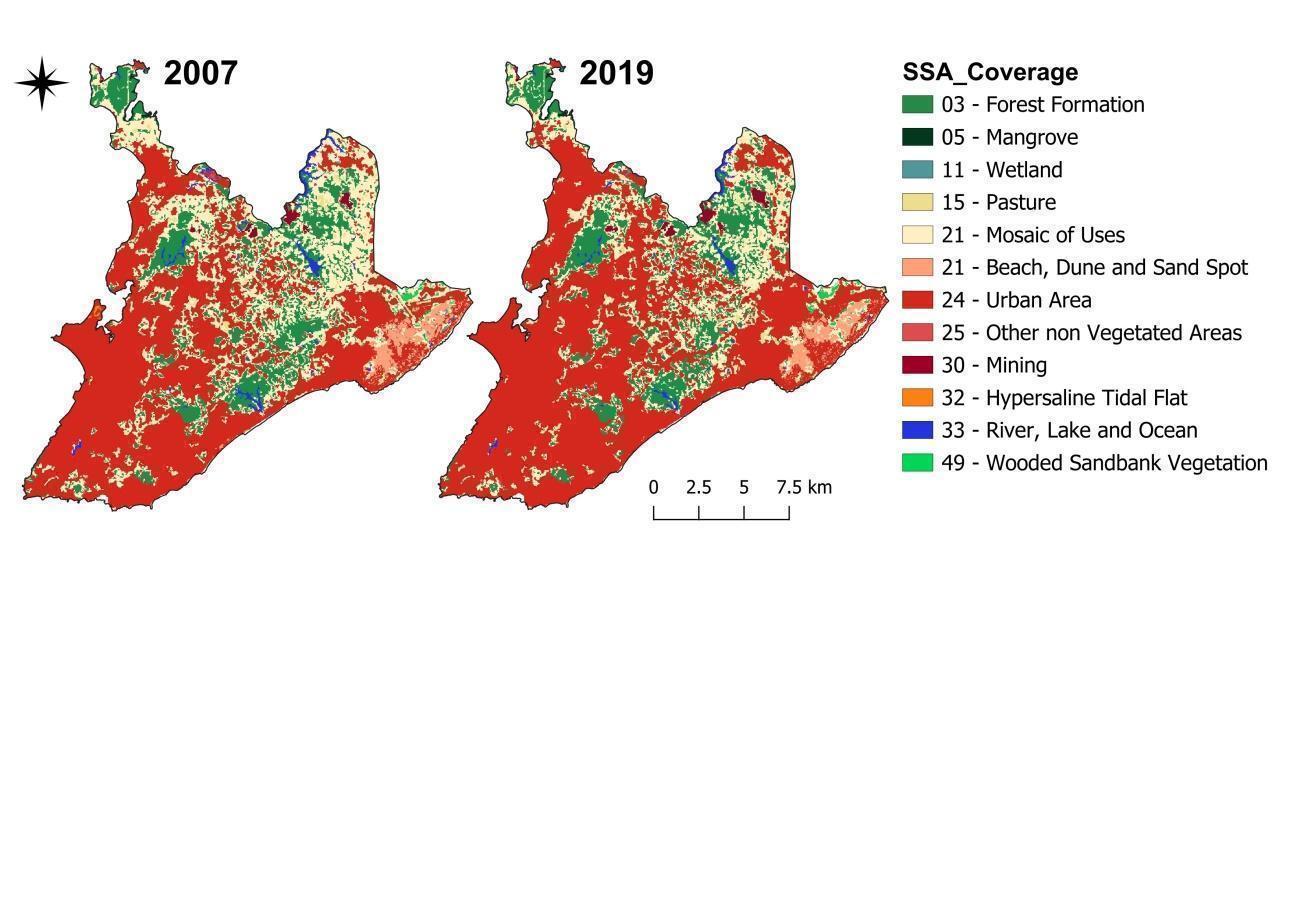


Legend: Land coverage graph of Salvador at 2007 and 2019. Data: MapBiomas, collection 8.0.

**Supplementary material 4. Figure S2: Marginal Posterior Distributions of Parameters from the Multivariate Model**
The figure shows the marginal posterior distributions of the main parameters in the multivariate model, including fixed effects, Forest area (Forest_are), Deforested area (Defor_are), and population size (Pop_1000), as well as random effects, illustrating the uncertainty and contribution of each predictor.


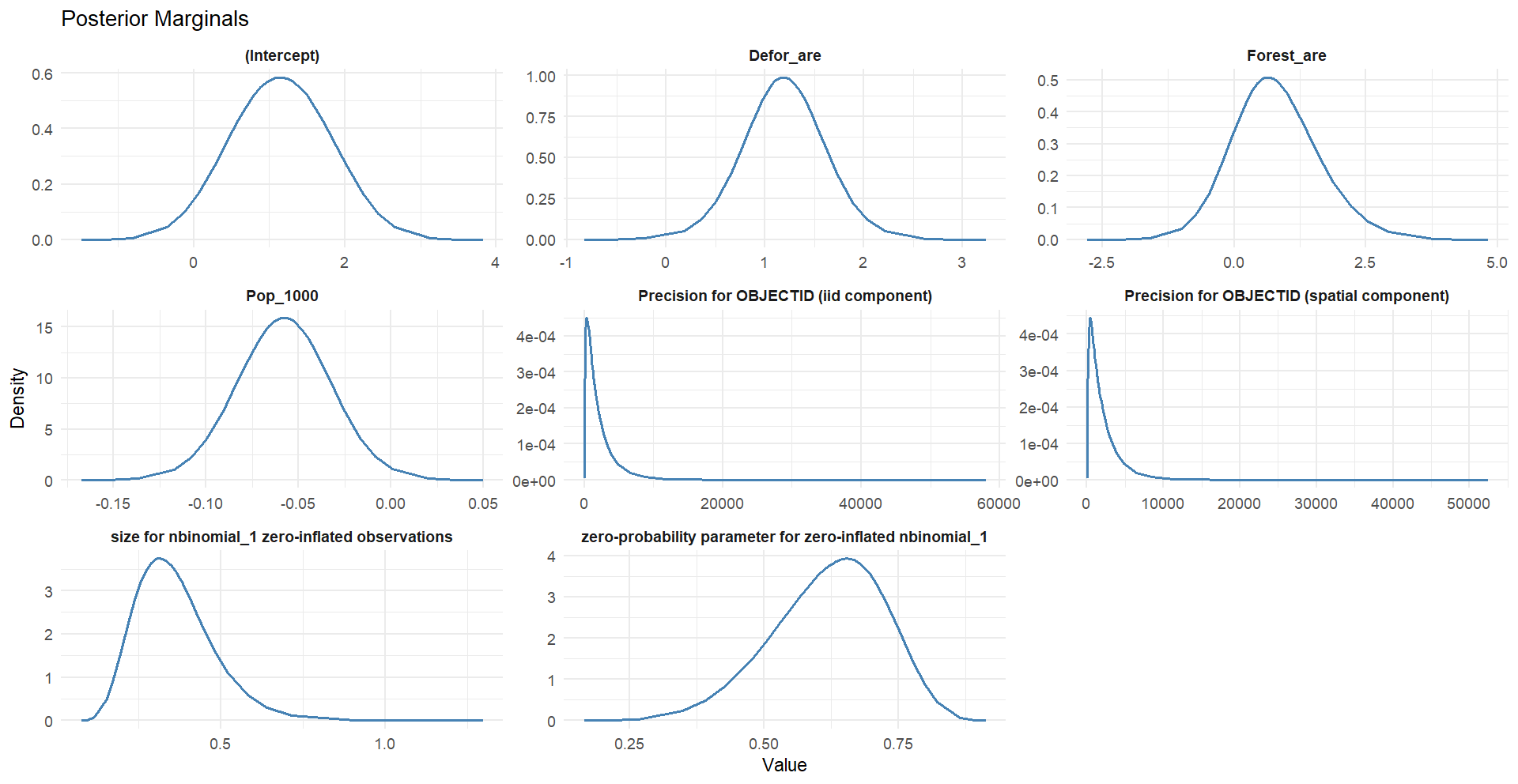


**Supplementary material 5 –** Dataset and variable codes.
